# Evaluation of Lung Functions, Blood Pressure, and Hearing Deterioration in Tile Setters

**DOI:** 10.7759/cureus.35250

**Published:** 2023-02-21

**Authors:** Vibha Gangwar, Nitin John, Manish Verma, Jyoti John, Rajani Bala Jasrotia, Amita Singh

**Affiliations:** 1 Department of Physiology, Dr Ram Manohar Lohia Institute of Medical Sciences, Lucknow, IND; 2 Physiology, All India Institute of Medical Sciences, Bibinagar, IND; 3 Biochemistry, All India Institute of Medical Sciences, Nagpur, IND; 4 Department of Physiology, All India Institute of Medical Sciences, Deoghar, IND

**Keywords:** crystalline silica dust, noise pollution index, hearing deterioration, altered blood pressure, compromised lung functions

## Abstract

Introduction: Tile setters in construction industries are exposed to a potent risk of silicosis as they are constantly exposed to crystalline silica dust during concrete finishing, cutting and fixing of the ceramic tiles. The noise produced by instruments used in tile settings may lead to noise-induced hearing loss. Noise above the permissible limit of 85 dB may lead to autonomic changes and alteration in blood pressure. These facts gave us an impetus to evaluate the pulmonary functions, blood pressure and hearing deterioration in tile setters and correlate these parameters for their present functional status with duration of exposure to tile cutting profession.

Methods: The pulmonary functions were evaluated with spirometer, autonomic status by recording the blood pressure and hearing loss by calculating the Hearing Deterioration Index (HDI) for hearing loss.

Results: There was significant decline in forced vital capacity (FVC), forced expiratory volume 1 sec (FEV1) and forced expiratory volume 1% (FEV1%) in tile setters. The decline in FVC, FEV1 and FEV1% was significantly higher in subjects with exposure of more than five years. There was increased systolic and diastolic blood pressure in those having more than five years of exposure in the tile setting profession as compared to less than five years. There was a positive correlation between years of service in the tile setting profession as well as sound exposure level with HDI and blood pressure in our subjects.

Conclusion: Prolonged exposure to the tile setting profession may lead to compromised lung function, hypertension and hearing deterioration in tile setters.

## Introduction

Tile setters in construction industries are exposed to a potent risk of silicosis given exposure to crystalline silica dust during concrete finishing, cutting, and fixing of the ceramic tiles [1,2]. The ceramic tiles are a constituent of clay, silica sand, stones of pottery, and talc. The dust of these materials, mainly clay and silica, can pose a health hazard to the lungs of patients. Those workers in mining industries are also exposed to the risk of pneumoconiosis and particularly silicosis. Many mines are functional in India mostly in the states of Jharkhand, West Bengal, Bihar, and Chhattisgarh, and hence occupational health and safety norms need to be in practice there. The American Conference of Governmental Industrial Hygienists (ACGIH) has also classified silica in group A2 as a probable carcinogen, therefore reduceing the threshold limit value (TLV) of crystalline silica from 0.1 mg/m3 in 1986 to 0.025 mg/m3 in 2006 [3]. The diseases, mainly pneumoconiosis, noise-induced hearing loss, and cardiorespiratory tuberculosis, are three prevalent diseases among South African mine workers [4]. The prevalence of occupation-induced hearing loss in low- and middle-income (LAMI) countries is another growing health issue in Africa and especially in South Africa. Pneumoconiosis and noise-induced hearing loss are of grave concern in mining industries of Africa and so also globally [4,5]. 

Tiles are used for flooring worldwide. The construction workers include tile setters who not only cut the tiles but fix them too. The perpetual health risk in these tile workers is a concern as many of the tile contractors are private small enterprises and generally lack health awareness regarding health risks and issues associated with their occupation [1]. Therefore research studies involving the assessment of all associated risk needs to be carried out for better occupational health and safety in these tile cutters.

The lung pathology in silicosis is due to the deposition of fine crystalline silica dust particles in the lungs. The size of this silica dust particle is usually around 10 μm or less in diameter. This silica dust deposit in the lungs may lead to chronic obstructive pulmonary disease (COPD) and interstitial lung disease. It may also cause chronic bronchitis, asthma, and pneumoconiosis [6]. Pulmonary complications by silicosis are well known in tile cutters but no studies have been conducted on them assessing the deterioration in the hearing index or any associated autonomic effect though they have the risk of developing hearing loss as they are constantly using hand grinder tools for tile cutting. These grinding tools as well as tile polishing tools were known to produce noise above the normal sound level of 85 dB. Constant exposure to critical noise levels over 85 dB over the years may lead to hearing loss [7,8]. Noise does impact our autonomic nervous system and noise exposure over the permissible limit may lead to hypertension [9]. The use of personal protective equipment for the prevention of respiratory infections and hearing loss is not yet in practice by small-time contractors and construction workers.

Most of the research has evaluated lung function in miners, but there is a paucity of data on the impact of years of exposure to the profession of tile cutting on pulmonary functions. There have also no studies been carried out earlier to evaluate hearing deterioration on exposure to tools being used in tile cutting and the impact of the noise level over arterial blood pressure. These facts gave us an impetus to evaluate the forced vital capacity (FVC) and forced expiratory volume 1% (FEV1%) for assessing pulmonary functions, Hearing Deterioration Index (HDI) for hearing loss, and arterial blood pressure (BP) for evidence of hypertension, if any, in the tile setters and correlate these parameters for their present functional status with a duration of exposure to tile cutting and setting profession. We hypothesize that apart from deterioration of pulmonary functions there might be the prevalence of progressive hearing loss and altered BP over the years of exposure. 

## Materials and methods

This study was conducted in the Department of Physiology at Dr. Ram Manohar Lohia Institute of Medical Sciences, Lucknow. A total of 50 adult males age 20-40 years participated in this study, out of which 25 male tile setters working in the construction unit of our expansion academic building at our institute were taken as cases, and 25 healthy males from supervisory and office staff were also recruited as controls in our study. The research project was started after obtaining approval from the Ethics and Research Committee via letter no 3097/RMLIMS/2018 dated 08.10.2018 with IEC No. 4/18. The details of the research protocol were explained to the participants and written consent was obtained from the participants. All participant tile setters selected for the study were non-smokers, non-alcoholic, neither had any clinical signs and symptoms of illness nor were on any placebo or treatment, after their detailed history and examination. Those having a history of persistent cough, chronic bronchitis, asthma, or persistent dyspnoea were excluded from the study. All controls were healthy individuals, being non-smokers and non-alcoholic, and had no clinical evidence of any illness. After recording the health profile, a detailed clinical examination was conducted, vitals were recorded and then the investigations were carried out for evaluating pulmonary functions. Pulmonary functions were recorded by using a computerized spirometer (Easy on-PC; ndd Medical Technologies, Zurich, Switzerland) as per the European Respiratory Society/American Thoracic Society Clinical Practice Guidelines [10]. The observed value of FVC, forced expiratory volume 1 sec (FEV1), and FEV1% were noted. The blood pressure (BP) was recorded using a mercury sphygmomanometer at the same time in both groups. BP was recorded in both arms at an interval of five minutes and an average of the four readings was taken as the blood pressure of the participants. The mean of systolic BP (SBP) and diastolic BP (DBP) were recorded [9]. 

Evaluation of Hearing Deterioration Index (HDI)

Noise-induced hearing loss can be measured by a formula-based method being used in research for assessing the HDI. The HDI in our subject was calculated using the prescribed formula:

HDI=10log_{10}\left [ \int_{0}^{t}10\tfrac{L}{20} dt\right ]

The ‘L’ denotes the average sound level (dB) of exposure in an individual, and the ‘t’ represents the time of years of exposure to the sound level ‘L’. We calculated the time of exposure using daily hours of working and it was multiplied by the year of exposure [8,9].

Recording of sound using sound level meter

The mean exposure to sound level was measured using a pocket type of sound level meter that has a measurement range between 30 to 130 dB. The sound level was recorded on three occasions for three consecutive days to know the average level of sound exposure in an eight-hour shift. The exposure level to sound was measured using International Electro-technical Commission (IEC) guidelines [11]. We also segregated the observed values of pulmonary functions, BP, and HDI in tile setters into two subgroups depending on the duration of exposure to the tile profession into less than five years and five or more years to compare the duration of exposure with the impact on pulmonary function profile, BP, and HDI. In addition, we also conducted a correlation analysis of years of service and sound exposure level with HDI and BP.

The statistical analysis was conducted using the software Statistical Package for Social Sciences (SPSS) version 22 (IBM Corp., Armonk, NY, USA). The mean values of FVC, FEV1%, FEV1, systolic and diastolic BP, and HDI were analyzed in the 25 tile setters and compared with that of 25 controls. P values less than 0.05 were considered significant. Pearson's correlation coefficient was employed to ascertain the correlation between the HDI and service years, hours of sound exposure level, SBP, and DBP.

## Results

In this study a total of 50 participants were enrolled, including 25 controls and 25 tile setters. As observed in Table 1, the pulmonary functions were found to be decreased in tile setters as compared to controls. There were significant declines in FVC, FEV1 and FEV1% in tile setters. 

**Table 1:** Mean values of FVC, FEV1, FEV1%, HDI, SBP and DBP in tile setters and controls. FVC = Forced Vital Capacity (liters), FEV1 = Forced Expiratory Volume 1 Sec (liters), FEV1% = Forced Expiratory Volume 1%, HDI = Hearing Deterioration Index, SBP = systolic blood pressure in millimeters of mercury, and DBP = diastolic blood pressure in millimeters of mercury

Parameter	Controls N=25		Tile Setters N=25		p-Value
	Mean	±SD	Mean	±SD	
Forced Vital Capacity (liters)	3.74	0.45	2.94	0.40	<0.001
Forced Expiratory Volume 1 Sec (liters)	3.15	0.40	2.42	0.42	<0.001
Forced Expiratory Volume 1%	84.01	1.21	81.80	4.09	0.013
Hearing Deterioration Index	33.53	1.99	50.52	4.43	<0.001
Systolic Blood Pressure (mmHg)	118.80	12.43	141.60	15.52	<0.001
Diastolic Blood Pressure (mmHg)	79.44	9.50	95.92	14.22	<0.001

Moreover on comparison of these values in relation to years of exposure to tile profession among subgroups, it was observed that the decline in FVC, FEV1 and FEV1% was significantly higher in subjects with five or more years of exposure than less than five years (Table 2).

**Table 2:** Mean values of FVC, FEV1, FEV1%, HDI, SBP and DBP in tile setters in reference to duration of exposure to tile setting profession. FVC = Forced Vital Capacity (liters), FEV1 = Forced Expiratory Volume 1 Sec (liters), FEV1% = Forced Expiratory Volume 1%, HDI = Hearing Deterioration Index, SBP = systolic blood pressure in millimeters of mercury, and DBP = diastolic blood pressure in millimeters of mercury

Parameter	Tile Setters N=13 Years of Exposure <5 years		Tile Setters N=12 Years of Exposure ≥5 years		P Value
	Mean	±SD	Mean	±SD	
Forced Vital Capacity (liters)	3.26	0.24	2.61	0.21	<0.001
Forced Expiratory Volume 1 Sec (liters)	2.76	0.21	2.06	0.24	<0.001
Forced Expiratory Volume 1%	84.72	0.97	78.65	3.82	<0.001
Hearing Deterioration Index	47.03	3.21	54.31	1.20	<0.001
Systolic Blood Pressure (mmHg)	128.31	5.88	156.00	7.29	<0.001
Diastolic Blood Pressure (mmHg)	84.00	7.53	108.83	5.42	<0.001

We also observed in Table 3 and Table 4 that with increase in the total service years there is significant increase in total duration of sound exposure in tile setters as compared to controls, and this may be a cause for loss of hearing.

**Table 3:** Mean value of total service years and duration of exposure to sound level in hours in tile setters and controls Tiles setters were exposed to an average sound level of 90 dB while controls were exposed to an average sound level of 55 dB.

Parameter	Controls N=25		Tile Setters N=25	
	Mean	±SD	Mean	±SD
Total Service Years	4.4800	2.43447	5.2600	3.97419
Total duration of Exposure to Sound level (hours)	13081.6	7108.67	17987.2	13645.47

**Table 4:** Correlation between total service years and sound exposure (hours) in controls and tile setters

Parameter	Karl-Pearson’s correlation coefficient (r- values)	p-Value
Total Service Years and Total Duration of Exposure (hours) in Controls	1.0	<0.001
Total Service Years and Total Duration of Exposure (hours)in Tile Setters	0.936	<0.001

**Figure 1:**
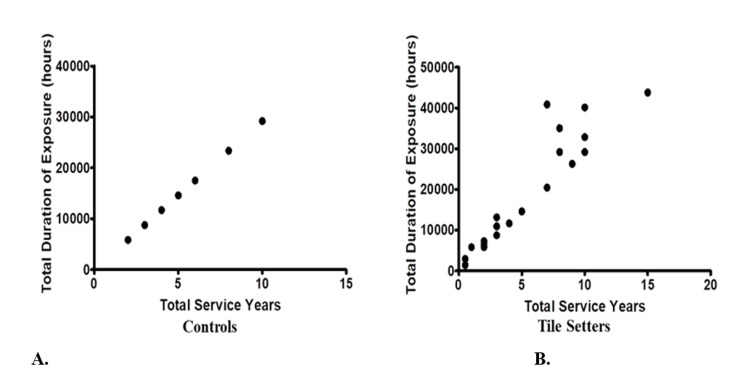
Graph showing correlation between total service years and total duration of sound exposure (in hours) in controls (A) and tile setters (B)

Figure 1 shows a graph between total duration of exposure and total service years in controls (Figure 1A), which is a straight line, on the other hand if we compare total duration of exposure and total service years in tile setters (Figure 1B), the curve is not a straight line, which indicates that tile setters are exposed to a higher level of sound with same years of service as compared to controls.

A positive correlation has been found, as shown in Table 5, between years of service in the tile setting profession as well as sound exposure level with HDI and BP indicating that apart from pulmonary function deterioration there is also risk of development of hearing loss and hypertension in tile setters.

**Table 5:** Mean value of total service years and duration of exposure to sound exposure levels 90 (dB) in hours in tile setters in reference to duration of years of exposure to tile setter profession.

Parameter	Tile Setters N=13 Exposure Duration <5 years		Tile Setters N=12 Exposure Duration ≥ 5 years		P Value
	Mean	±SD	Mean	±SD	
Total Service Years	1.96	1.11	8.83	2.52	<0.001
Duration of Exposure to Sound Exposure Levels 90 (dB) in hours	6738.5	3664.01	30173.0	8947.86	<0.001

Table 6 shows the significant correlation between years of service and hours of sound exposure with HDI. There are significants correlation of systolic and diastolic BP with hours of sound exposure in tiles setters.

**Table 6:** Correlation between years of service and sound exposure level with HDI and blood pressure in tile setters HDI = Hearing Deterioration Index, SBP = systolic blood pressure in millimeters of mercury, and DBP = diastolic blood pressure in millimeter of mercury, Significant (p<0.05)

Parameters	Karl-Pearson’s correlation coefficient (r-value)	p-Value
HDI and Service Years	0.913	<0.001
HDI and Sound Exposure (hours)	0.879	<0.001
SBP and Sound Exposure (hours)	0.941	<0.001
DBP and Sound Exposure (hours)	0.880	<0.001

**Figure 2:**
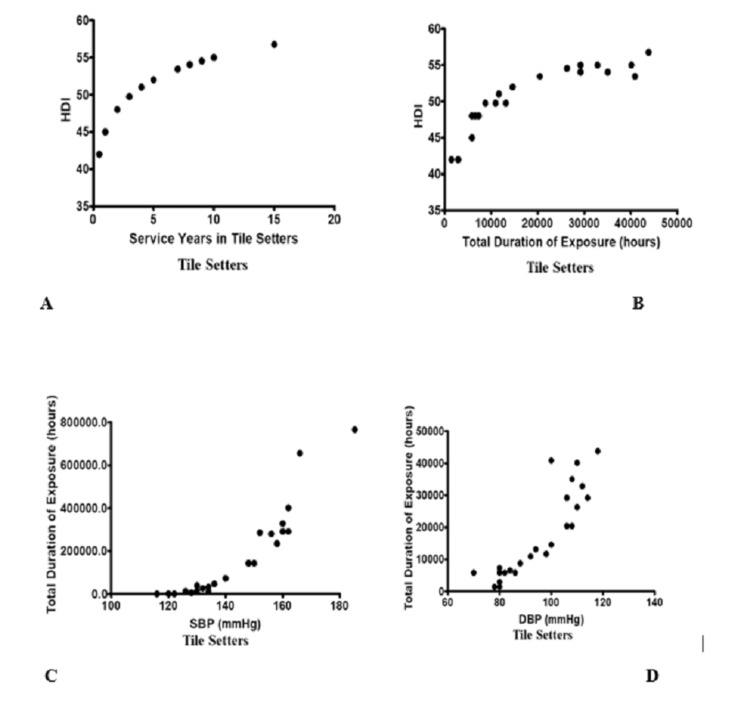
Correlation between years of service and HDI (A), total duration of sound exposure in hours with HDI (B), SBP in millimetres of mercury and total duration of exposure in hours (C), DBP in millimetres of mercury and total duration of exposure in hours (D) in tiles setters. HDI = Hearing Deterioration Index, SBP = systolic blood pressure in millimetres of mercury, and DBP = diastolic blood pressure in millimetres of mercury

Figure 2 shows a graph of correlation between years of service and HDI (Figure 2A), and total duration of sound exposure in hours with HDI (Figure 2B) in tile setters. We can observe that with increasing years of service and total duration of sound exposure, HDI increases in tile setters due to noise-induced hearing loss. A graph of correlation between SBP mercury and total duration of exposure (Figure 2C), and DBP and total duration of exposure (Figure 2D) in tile setters. Both blood pressures show a sharp increase and fluctuations with increase in total duration of sound exposure.

## Discussion

Noise impact on human health has been a health concern globally and there are increase incidences of occupation-based noise-induced hearing loss [12]. The permissible noise exposure level is around 85 dB in an eight-hour shift. Exposure to noise over 85 dB over the years leads to release of stress hormones in the body. The increased circulatory epinephrine levels may lead to hypertension and other cardiovascular disorders, the global burden of occupational noise-induced hearing loss [13-15].

Decline in lung functions is due to exposure to silica dust while tile setting in construction work. Our findings are in concurrence with Tavakol et al., as noted by them in construction workers more than half of these workers (51.8%) were having restrictive pulmonary disorders while a few (around 4.70%) had obstructive disorders [16]. Exposure over years to silica dust may lead to deposit of the smaller particulates in the alveoli and can lead to chronic bronchitis, COPD and interstitial lung diseases. 

Calvert et al. in their studies also found a stronger association between exposure to crystalline silica and silicosis, lung cancer, COPD, and pulmonary tuberculosis [17]. These silica particulates when deposited initiates in lungs lead to a chronic inflammatory response in the lung parenchyma and over the years may lead to lung fibrosis and restrictive lung disease.

The development of silicosis is never acute in onset but it occurs gradually over the years that alters the lung structure and its constituents, leading to fibrosis and decrement in the normal lung expansion thereby reducing the lung volume and capacity. Silicosis eventually leads development of nodular pulmonary fibrosis [18]. We also found in the study that the decrement in lung function was more in those exposed to the tile profession for five or more years (FEV1%=78.65) than those of less than five years (FEV1%=84.72).

We have observed that the average noise exposure in an eight-hour shift was around 90 dB in tile setters. The instruments used, like angle grinders, grinding machines and ceramic tile cutters, all produce noises. The close proximity of these instruments to the ears of the tile-setting workers leads to noise exposure. The increased stress hormones will also increase anxiety, lead to sleeplessness, sleep disorders and hypertension. 

The increased systolic and diastolic blood pressure in subjects recruited in our study is attributable to noise-induced cardiovascular effects and continuous exposure over a duration of years has led to development of hypertension in our subjects, particularly in individuals having five or more years of exposure in the tile setting profession. Our findings are in concurrence with those of Edwards et al. who found that nearly 66.7% of the mining workers sampled in the first year and 78.4% in the second year were exposed to noise levels of more than 85 dB [19].

This impact of noise-induced hearing loss is also observed in our study and is evident from the increased HDI from 42 dB to 55 dB approximately within 20 years of exposure in our tile setters. Moreover we have also observed a positive correlation between years of service and sound exposure level with HDI and BP. These findings of our study have been noticed in tile setters but similar HDI index measured by Sogebi et al. in the general population and Balaji et al. in bus drivers have also found a co-relation over years of sound exposure to increase in HDI [7,8].

The novelty in our study was the HDI calculation in tile setters which has not been carried out in India yet as well as there is global paucity of similar studies being conducted. Moreover correlation analysis of years of service in the tile setting profession and sound exposure level with HDI and BP which will alert the occupational health and safety officers to be vigilant in employing suitable preventive measures in the construction industry. 

The limitation of our study was the smaller sample size and we recommend that future studies be conducted with a larger population in the profession and the regular complete health profile be investigated in tile setters so that adverse health effects can be prevented.

Employing safety measures in the construction industry, following wet grinding in tile setters, and use of protective masks and ear plugs will help to reduce the impact of the profession on lungs, hearing, and blood pressure [20].

## Conclusions

There was a significant decline in FVC, FEV1, and FEV1% in tile setters. The decline in FVC, FEV1, and FEV1% was significantly higher in subjects with five to 10 years of exposure than in those with zero to five years. We also observed that with an increase in the total service years, there is an increase in total hours of sound exposure in tile setters and this may lead to deterioration in hearing. There was increased systolic and diastolic blood pressure in our subjects particularly in those having five to 10 years of exposure in the tile setting profession as compared to one to five years of exposure. There was a positive correlation between years of service in the tile setting profession as well as sound exposure level with HDI and BP in our subjects.
